# Methamphetamine-Induced Sleep Impairments and Subsequent Slow-Wave and Rapid Eye Movement Sleep Rebound in Male Rhesus Monkeys

**DOI:** 10.3389/fnins.2022.866971

**Published:** 2022-04-07

**Authors:** Laís F. Berro, John S. Overton, James K. Rowlett

**Affiliations:** Department of Psychiatry and Human Behavior, Center for Innovation and Discovery in Addictions, University of Mississippi Medical Center, Jackson, MS, United States

**Keywords:** methamphetamine, sleep, EEG, telemetry, rhesus monkey, rebound, REM

## Abstract

Use of amphetamine-type stimulants is associated with numerous adverse health outcomes, with disturbed sleep being one of the most prominent consequences of methamphetamine use. However, the extent to which methamphetamine alters sleep architecture, and whether methamphetamine-induced sleep impairment is associated with next-day sleep rebound effects, has received relatively little investigation. In the present study, we investigated the effects of acute morning methamphetamine administration on sleep parameters in adult male rhesus monkeys (*N* = 4) using a fully-implantable telemetry system. Monkeys were prepared with telemetry devices that continuously monitored electroencephalography (EEG), electromyography (EMG) and electrooculography (EOG) throughout the night. We investigated the effects of morning (10h00) administration of methamphetamine (0.01–0.3 mg/kg, i.m.) on sleep during the night of the injection. In addition, we investigated sleep during the subsequent night in order to assess the possible emergence of sleep rebound effects. Methamphetamine administration dose-dependently increased sleep latency and wake time after sleep onset (WASO). Methamphetamine also decreased total sleep time, which was reflected by a decrease in total time spent in N2, slow-wave (N3) and REM sleep stages, while increasing the percentage of total sleep time spent in sleep stage N1. Importantly, methamphetamine decreased time spent in N3 and REM sleep even at doses that did not significantly decrease total sleep time. Sleep rebound effects were observed on the second night after methamphetamine administration, with increased total sleep time reflected by a selective increase in time spent in sleep stages N3 and REM, as well as a decrease in REM sleep latency. Our findings show that methamphetamine administered 8 h prior to the inactive (dark) phase induces marked changes in sleep architecture in rhesus monkeys, even at doses that do not change sleep duration, and that sleep rebound effects are observed the following day for both N3 and REM sleep stages.

## Introduction

Amphetamine-type stimulants can be useful therapeutic tools for the treatment of a variety of health conditions, from sleep-related disorders such as narcolepsy and hypersomnia to attention deficit/hyperactivity disorder and obesity (McElroy, 2017; Bassetti et al., 2019; Posner et al., 2020; Trotti and Arnulf, 2021). The most recent National Surveys on Drug Use and Health (2015–2016) reported that nearly 6.6% (∼16 million) of United States adults used prescription stimulants during those years (Compton et al., 2018). However, the clinical utility of stimulants is limited by the side effects associated with the use of these drugs, including liability for abuse. Data from 2018 show that more than 5 million United States individuals 12 and older reported past-year misuse of prescription stimulants, and past-year methamphetamine use in particular increased from 1.4 million in 2016 to 1.9 million in 2018 (Substance Abuse and Mental Health Services Administration [SAMSHA], 2019). Of note, emergency department visits associated with amphetamine-type stimulant-induced overdoses also have increased in recent years (Vivolo-Kantor et al., 2020).

Use and abuse of amphetamine-type stimulants is associated with numerous adverse health outcomes (Herbeck et al., 2015; Rommel et al., 2015), with disturbed sleep being one of the most prominent consequences of acute and chronic stimulant use (Cruickshank and Dyer, 2009). Herrmann et al. (2017) showed that morning administration of oral methamphetamine disrupted polysomnography-based sleep parameters in recreational stimulant users, increasing latency to fall asleep and decreasing sleep efficiency. Corroborating these findings, we have shown previously that morning administration of methamphetamine disrupts actigraphy-based sleep parameters in rhesus monkeys, increasing sleep latency and decreasing sleep efficiency both in naïve monkeys (Berro et al., 2021a) and in monkeys with a chronic history of methamphetamine intake (Berro et al., 2016, 2017a,b). Importantly, Herrmann et al. (2017) also showed that methamphetamine dose-dependently decreased time in sleep stage N2 and in rapid eye movement (REM) sleep, as well as the number of REM sleep episodes.

In a recent and thorough review of the literature by Vrajová et al. (2021), it was noted that research on methamphetamine-induced sleep disruption in non-human primates is at preliminary stages and has relied mostly on actigraphy-based sleep measures. Therefore, studies investigating more direct measures, such as EEG-based sleep, are necessary in order to more rigorously evaluate changes in sleep (i.e., sleep architecture) following methamphetamine administration in non-human primates. Sleep architecture refers to the structure of sleep cycles throughout the night and the four sleep stages, non-rapid eye movement (NREM) sleep stages N1, N2, and N3 (also known as slow-wave sleep), as well as REM sleep (Berry et al., 2018). Measuring sleep architecture in the context of drug administration is important for evaluating whether drugs affect the distribution and density of sleep stages throughout the night, as well as REM sleep rhythmicity, in addition to sleep duration. Moreover, the extent to which methamphetamine-induced sleep impairment is associated with next-day sleep rebound effects has not been investigated. In the present study we evaluated the effects of acute morning methamphetamine administration on “polysomnography”-based sleep parameters in male rhesus monkeys using a fully-implantable telemetry system that allows for the continuous recording of electroencephalography (EEG), electromyography (EMG) and electrooculography (EOG). We also evaluated sleep parameters during the second night following methamphetamine administration in order to assess whether methamphetamine-induced REM sleep suppression would be associated with the emergence of REM sleep rebound and/or other compensatory changes on sleep architecture.

## Materials and Methods

### Subjects

Four adult (ages 9–15) male rhesus monkeys (*Macaca mulatta*) weighing 12–16 kg were housed individually, but had visual, auditory and olfactory contact with other monkeys throughout the study, as well as access to chew toys and a mirror in their cage. Subjects were maintained on a 12 h light/12 h dark cycle (lights on at 06.00, lights off at 18.00), at a temperature of 21 ± 2°C. Water was available *ad libitum* and monkey diet available once/day, supplemented by fresh fruit and forage. Monkeys were weighed monthly during physical examinations, and amount of chow for each monkey was determined in consultation with veterinary staff to be that which maintains healthy weights in rhesus monkeys. All four subjects participated in a previous study investigating the effects of acute benzodiazepine administrations (Berro et al., 2021b). Animals had been previously exposed to five acute benzodiazepine administrations at least 2 days apart, with the last administration being conducted at least 3 months prior to the beginning of the current study (Berro et al., 2021b). Therefore, the animals’ previous experimental history likely did not contribute to the present findings. All procedures and animal maintenance were in accordance with the Guide for the Care and Use of Laboratory Animals (National Research Council, 2011), and were reviewed and approved by the Institutional Animal Care and Use Committee of the University of Mississippi Medical Center.

### Surgical Procedures

The monkeys were prepared with a telemetry implant according to the surgical procedures described in detail by Berro et al. (2021b) at least 1 month prior to the beginning of tests. Briefly, the fully implantable telemetry devices [Model L04 PhysioTelTM Digital; Data Sciences International^®^ (DSI), St-Paul, MN] consisted of an implant device (implant weight: 56 g; implant volume: 29cc; implant dimensions: 59 × 38 × 15 mm) attached to a small antenna and to 8 leads, 2 for each biopotential channel (4 channels total). For the device implantation, a skin incision was made lateral to the spine beginning just caudal to the scapulae and extending caudally, and the device was placed under the latissimus dorsi muscle. A trocar and cannula then guided the antenna in a straight path distally from the device, and the biopotential leads from the dorsal pocket to a cranial incision. Four EEG leads (2 channels, 2 leads each) were placed in the skull, one in a central derivation (C4-Cz) and the other in an occipital derivation (O2-Oz). Scalp electrode placement was based on the human 10–20 System of Electrode Placement. Leads were secured to the skull with a stainless steel self-tapping round-head screw (Plastic One, VA, United States). The screws were attached to holes drilled through the bone, however the hole did not perforate the dura, allowing for supradural recording. All exposed wire and screws were covered with dental acrylic (Maxcem EliteTM, Kerr Corporation, CA, United States). For the placement of the EMG leads (1 channel, 2 leads), the cranial skin incision was extended as needed, ∼4 cm, to expose the longitudinal muscle of the neck (trapezius). Exposed wire leads were attached in a parallel manner to the neck muscle and secured in place using non-absorbable suture. For the placement of the EOG leads (1 channel, 2 leads), the leads were routed subcutaneously from the head incision to the eye orbit, exiting the eye orbit from underneath the upper eyelid. Exposed wire leads were then attached to and under the periosteum of the orbit (one adjacent to the lateral canthus of the eye and the other adjacent to the medial canthus of the eye) with non-absorbable suture.

### Experimental Design

The acute effects of daytime (10.00) administration of methamphetamine (0.03, 0.1, and 0.3 mg/kg, i.m.) and vehicle (saline) on EEG/EMG/EOG-based sleep were evaluated. Doses for this study were chosen initially based on prior experiments in which actigraphy-based sleep disruption was observed following i.v. self-administration of 0.36 to 1.38 mg/kg of methamphetamine (Berro et al., 2017a,b). These doses represent “total dose”, i.e., mg/kg dose consumed over a 1-h session, and were obtained with methamphetamine-experienced monkeys. Therefore, the doses for the present study were adjusted to a lower range out of safety concerns.

Telemetry implants were turned on at 17.00 (1 h before “lights off”) and recorded for a total of at least 14 h (until at least 07.00, 1 h after “lights on”) during the night of morning injections and also during the night following treatments. Implants were turned on by touching the area on the animal’s back where the telemetry device was implanted with a magnet while the receivers located inside the room were actively communicating with a computer located outside of the room. Before the beginning of experiments, animals were trained with positive reinforcement techniques to be touched on their back/dorso (telemetry device implant site). Our previous studies using actigraphy showed no significant differences in daytime activity on intervening days (between methamphetamine self-administration sessions) and baseline days (Berro et al., 2017a). Therefore, we did not conduct daytime telemetry monitoring in the present study. All recordings were conducted in the animals’ home cages. Two telemetry receivers (TRX-1; DSI, St. Paul, MN, United States) were mounted inside the room where animals were housed. Continuous acquisition of the EEG/EMG/EOG signals occurred using the Ponemah physiologic data acquisition software (version 6.41; DSI, St. Paul, MN, United States). Test sessions were performed on Mondays and Thursdays, allowing for a 2-day washout period to elapse between each treatment and for the evaluation of next-day rebound effects. A baseline sleep recording was conducted both before and after the end of the test sessions, and baseline sleep data for each monkey consist of an average of the two baseline recording sessions. The order of tests (including drug doses and vehicle) was randomized but counterbalanced across subjects.

### Sleep-Wake State Analysis

Sleep-wake states were determined by visual inspection of EEG, EMG and EOG signals using NeuroScore software (version 3.2.0; DSI, St. Paul, MN, United States) according to the American Academy of Sleep Medicine (AASM) guidelines for adult human sleep scoring (Manual version 2.5; Berry et al., 2018), adapted for rhesus monkeys (Berro et al., 2021b). Sleep-wake state scoring was conducted based on recordings from the central derivation (C4-Cz). Recordings from leads placed centrally reflect EEG activity summed from both frontal and parietal regions and are considered the most sensitive for recording sleep-related activity, with a single central derivation not differing from the AASM recommended EEG derivations in terms of scoring reliability (Ruehland et al., 2011). Trained investigators classified each 30 s epoch as one of five states: WAKE, non-rapid eye movement (NREM) sleep stages N1, N2, or N3, or rapid eye movement (REM) sleep. WAKE was scored when > 50% of the epoch consisted of alpha (8–13 Hz) activity or low amplitude, mixed frequency (2–7 Hz) activity and active EMG, possibly accompanied by EOG reflecting rapid eye movements and/or rapid eye blinks. N1 (Stage 1) was scored when 50% of the epoch consisted of relatively low amplitude, mixed frequency (2–7 Hz) activity and <50% of the epoch contained alpha (8–13 Hz) activity accompanied with lower EMG activity, and possible presence of slow eye movements. N2 (Stage 2) was scored when K-complexes (1–2 Hz isolated waves) and/or sleep spindles (regular 12–16 Hz EEG sequences) were observed and < 20% of the epoch contained high amplitude (>75 μV), low frequency (1–4 Hz) activity. N3 was scored when ≥ 20% of the epoch consisted of high amplitude (>75 μV), low frequency (1–4 Hz) EEG waves (i.e., slow wave activity). REM was scored when the epoch contained relatively low voltage, mixed frequency activity with predominant theta activity (4–8 Hz) accompanied with low EMG activity (i.e., atonia) and EOG showing rapid eye movements. Epochs with prominent artifacts were identified and excluded from scoring.

In addition to the distribution of sleep stages throughout the night, other measures of interest gathered from the sleep-wake scoring were sleep latency (time to fall asleep, in minutes, from “lights off”), REM sleep latency (time to first REM sleep episode, in minutes, from “lights off”), total sleep time (time spent in any sleep stage, in minutes, from “lights off” to “lights on”) and wake time after sleep onset (WASO, time spent in the WAKE stage, in minutes, after first sleep epoch until “lights on”).

### Data Analysis

Sleep data were analyzed using separate one-way repeated-measures (RM) analysis of variance (ANOVA) with treatment as the factor. Sleep data on nights of methamphetamine treatment were compared to sleep data on nights of vehicle treatment. “Following night” sleep data (rebound effects) were compared to baseline sleep data. Multiple comparisons were conducted using Bonferroni t-tests. All graphical data presentations were created and all statistical tests were performed using GraphPad Prism 9 (GraphPad Software, vers. 9.1.2). Significance was accepted at an alpha of *p* ≤ 0.05.

## Results

### General Sleep Parameters

**FIGURE 1:**
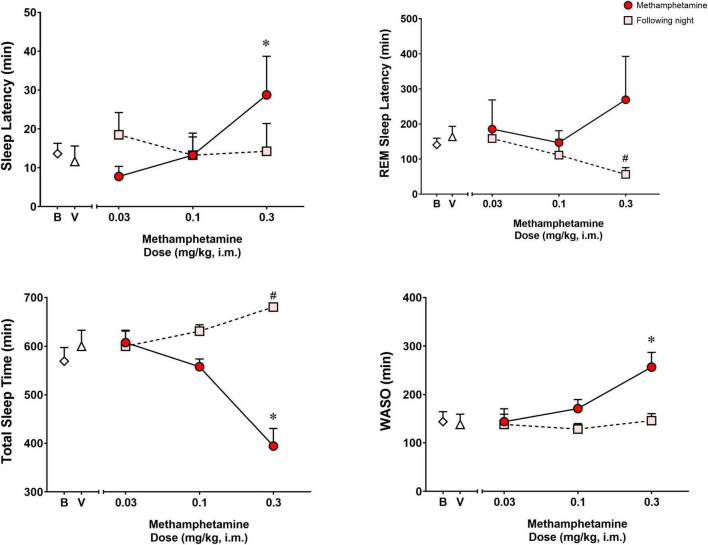
Sleep latency, rapid eye movement (REM) sleep latency, total sleep time and wake time after sleep onset (WASO) on the night of methamphetamine treatments and during the following night (second night after methamphetamine treatments) in male rhesus monkeys (*N* = 4). Sleep data on nights of methamphetamine treatment were compared to sleep data on nights of vehicle (V) treatment. “Following night” sleep data (rebound effects) were compared to baseline (B) sleep data. Data are expressed as mean ± SEM. *p < 0.05 compared to vehicle (V); #*p* < 0.05 compared to baseline (B).

Figure 1 shows the effects of vehicle or methamphetamine treatment on sleep latency, REM sleep latency, total sleep time and WASO, as well as sleep parameters during the following night (second night after methamphetamine treatments). Significant differences were observed with treatments (vehicle vs. methamphetamine doses) for sleep latency [*F*(3,9) = 4.485, *p* < 0.05], total sleep time [*F*(3,9) = 13.55, *p* < 0.01] and WASO [*F*(3,9) = 10.15, *p* < 0.01], but not for REM sleep latency [*F*(3,9) = 0.5665, *p* = 0.65]. Bonferroni *t*-tests showed that the highest dose of methamphetamine (0.3 mg/kg) significantly increased sleep latency and WASO, while decreasing total sleep time (*p* < 0.05 for all measures).

Significant differences also were observed between baseline and the following night sleep parameters for REM sleep latency [*F*(3,9) = 4.907, *p* < 0.05] and total sleep time [*F*(3,9) = 6.718, *p* < 0.05], with previous day treatment with the highest dose of methamphetamine significantly decreasing latency to first REM sleep epoch and increasing total sleep time (*p*’s < 0.05, Bonferroni *t*-tests). No significant differences were found between baseline sleep and the following night sleep for sleep latency [*F*(3,9) = 0.2316, *p* = 0.87] or WASO [*F*(3,9) = 0.9720, *p* = 0.44].

### Distribution of Sleep Stages Across the Night

**FIGURE 2:**
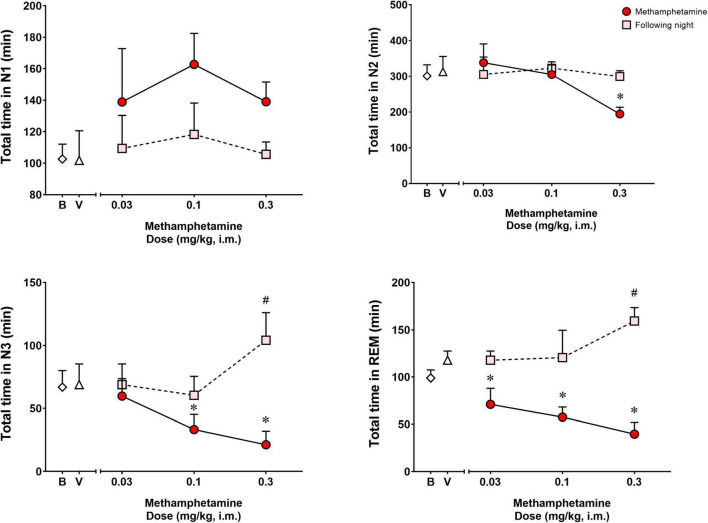
Total time spent in sleep stages N1, N2, N3, and REM on the night of methamphetamine treatments and during the following night (second night after methamphetamine treatments) in male rhesus monkeys (*N* = 4). Sleep data on nights of methamphetamine treatment were compared to sleep data on nights of vehicle (V) treatment. “Following night” sleep data (rebound effects) were compared to baseline (B) sleep data. Data are expressed as mean ± SEM. *p < 0.05 compared to vehicle (V); #*p* < 0.05 compared to baseline (B).

Figure 2 shows time spent in sleep stages N1, N2, N3, and REM during the night of vehicle or methamphetamine treatments, and during the following night (second night after methamphetamine treatments). Significant differences were observed with treatments (vehicle vs. methamphetamine doses) for total time spent in sleep stage N2 [*F*(3,9) = 5.748, *p* < 0.05], N3 [*F*(3,9) = 12.27, *p* < 0.01] and REM [*F*(3,9) = 11.52, *p* < 0.01], but not sleep stage N1 [*F*(3,9) = 2.577, *p* = 0.11]. Bonferroni *t*-tests showed that the highest dose of methamphetamine (0.3 mg/kg) significantly decreased time spent in sleep stage N2 (*p* < 0.05). Methamphetamine administration at the dose of 0.1 and 0.3 mg/kg significantly decreased time spent in sleep stage N3 (*p*’s < 0.05, Bonferroni *t*-tests). Significant decreases in REM sleep duration also were observed after administration of all doses of methamphetamine compared to vehicle (*p*’s < 0.05, Bonferroni *t*-tests).

Significant differences were observed between baseline and the following night sleep parameters for total time spent in sleep stages N3 [*F*(3,9) = 9.373, *p* < 0.01] and REM [*F*(3,9) = 4.391, *p* < 0.05], but not sleep stages N1 [*F*(3,9) = 0.4262, *p* = 0.73] and N2 [*F*(3,9) = 0.1669, *p* = 0.91]. Bonferroni *t*-tests showed that previous day treatment with the highest dose of methamphetamine (0.3 mg/kg) led to significant increases in time spent in N3 and REM compared to baseline (*p* < 0.05 for both measures).

**FIGURE 3:**
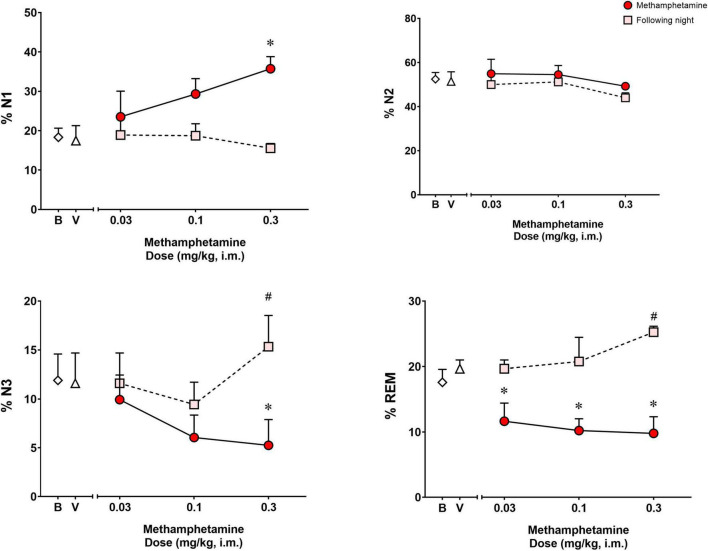
Percentage (%) of total sleep time spent in sleep stages N1, N2, N3, and REM on the night of methamphetamine treatments and during the following night (second night after methamphetamine treatments) in male rhesus monkeys (*N* = 4). Sleep data on nights of methamphetamine treatment were compared to sleep data on nights of vehicle (V) treatment. “Following night” sleep data (rebound effects) were compared to baseline (B) sleep data. Data are expressed as mean ± SEM. *p < 0.05 compared to vehicle (V); #*p* < 0.05 compared to baseline (B).

Analysis of the percentage of the total sleep time spent in each sleep stage (Figure 3) during the night of methamphetamine treatments showed significant differences for % N1 [*F*(3,9) = 6.391, *p* < 0.05], % N3 [*F*(3,9) = 5.150, *p* < 0.05] and % REM [*F*(3,9) = 7.251, *p* < 0.01], but not % N2 [*F*(3,9) = 0.8404, *p* = 0.50]. Bonferroni *t*-tests showed that the highest dose of methamphetamine (0.3 mg/kg) increased % N1 and decreased % N3 (*p* < 0.05 for both measures). Significant decreases in % REM also were observed after administration of all doses of methamphetamine compared to vehicle (*p*’s < 0.05, Bonferroni *t*-tests).

Figure 3 illustrates the percentage of the total sleep time spent in each sleep stage during the following night (second night after methamphetamine treatments). Significant differences were observed for % N3 [*F*(3,9) = 3.968, *p* < 0.05] and % REM [*F*(3,9) = 5.902, *p* < 0.05], but not % N1 [*F*(3,9) = 0.8035, *p* = 0.523] or % N2 [*F*(3,9) = 1.609, *p* = 0.25]. Methamphetamine administration at the highest dose (0.3 mg/kg) during the previous day led to a significant increase in % N3 compared to baseline (*p* < 0.05, Bonferroni *t*-tests). Significant increases in % REM also were observed during the following night after administration of all doses of methamphetamine compared to baseline (*p* < 0.05, Bonferroni *t*-tests).

## Discussion

In the present study, we used telemetric EEG/EMG/EOG recordings to determine sleep patterns of adult rhesus monkeys during the 12 h dark phase, and the effects of methamphetamine administration on sleep parameters. Specifically, we obtained full-night sleep parameters in rhesus monkeys under our experimental conditions using fully-implantable, wireless telemetry devices that allow for recording of EEG/EMG/EOG in freely-moving animals in their home-cages. Previous studies have characterized sleep-wake patterns in rhesus (Hsieh et al., 2008) and cynomolgus macaques (Authier et al., 2014; Rachalski et al., 2014; Goonawardena et al., 2018) using similar procedures. While consistent findings were obtained in terms of sleep stage distribution across the night between our study under baseline conditions and that by Hsieh et al. (2008), the latter authors used a 16:8 light-dark cycle, in contrast to a 12:12 light-dark cycle used in the present study. Importantly, Hsieh et al. (2008) found that rhesus monkeys under a light-dark cycle allowing only 8 h of dark phase exhibited frequent daytime napping, especially in the latter part of the day. While we did not evaluate daytime napping in the present study, previous reports from our laboratory using actigraphy show that rhesus monkeys generally do not exhibit nap bouts during the daytime when under a 12:12 light-dark cycle (Berro et al., 2017a), which is corroborated by studies using telemetry-based EEG/EMG recordings in cynomolgus monkeys (Goonawardena et al., 2018). Of note, the distribution of sleep stages obtained in our 12 h baseline sleep recordings was very similar to that previously reported in adult rhesus monkeys during the dark phase using telemetry devices, although not a fully-implantable system (Daley et al., 2006). In this regard, the proportion of total sleep spent in the various stages of sleep in the study by Daley et al. (2006) was 10.8% N1, 56.4% N2, 20% N3, and 12.7% REM, compared to 18.3% N1, 52.4% N2, 11% N3, and 17.6% REM in the present study under baseline conditions. These proportions are very similar to those previously reported in adult healthy human volunteers: 9.7% N1, 50.6% N2, 19.5% N3, and 19.2% REM (Boulos et al., 2019). Overall, these studies show that rhesus monkeys are an excellent model organism for studying sleep due to the similarity in sleep architecture with humans, and exhibit consistent sleep distribution even across laboratories and using manual/visual sleep scoring techniques.

Of note, the studies described above investigating sleep patterns in non-human primates were conducted in adult rhesus monkeys, with ages ranging from 5 to 20 years (Daley et al., 2006; Berro et al., 2017a; Goonawardena et al., 2018). While it is well known from the human literature that aging can influence sleep duration, particularly REM sleep duration (Roffwarg et al., 1966), at least up until 15 years of age we do not see significant changes in sleep architecture with rhesus monkeys (results from the present study), and up to 20 years old, no age-related changes in actigraphy-based sleep have been reported (Berro et al., 2021b). We have previously reported that one 25 year old monkey in our colony showed longer baseline sleep latency and shorter baseline sleep duration compared to other monkeys (subject 98-003, Berro et al., 2021a). However, other monkeys in our colony, as young as 9 years old, also show shorter sleep duration (Berro et al., 2021a, 2022), demonstrating that, like humans, the sleep patterns of non-human primates may vary greatly between individual subjects.

This study shows full-night EEG/EMG/EOG-based sleep parameters following acute methamphetamine administered 8 h before the dark phase in primates, presumably when methamphetamine has been mostly or entirely metabolized. Our results show that morning methamphetamine administration dose-dependently increased sleep latency and WASO. Methamphetamine also decreased total sleep time, which was reflected by a decrease in total time spent in sleep stages N2, slow-wave (N3) and REM, while increasing the percentage of total sleep time spent in sleep stage N1. Importantly, methamphetamine decreased time spent in N3 and REM sleep even at doses that did not significantly decrease total sleep time. Of note, the overall effects of methamphetamine reported in this study are consistent with those previously reported under similar conditions, but using actigraphy-based sleep, in our laboratory (Berro et al., 2021a). In both studies, only the dose of 0.3 mg/kg of methamphetamine significantly increased sleep latency and decreased sleep efficiency (which can be inferred from the present study by the equation: total sleep time/12 h*100). Our actigraphy-based sleep study showed a ∼700 ± 412% increase in sleep latency and a ∼24 ± 4% decrease in sleep efficiency following 0.3 mg/kg methamphetamine administration compared to baseline (Berro et al., 2021a), and our current data show a 150 ± 83% increase in sleep latency and a 30 ± 7% decrease in sleep efficiency compared to baseline. Different subjects were used in the two studies, which could explain some of the variability between the two data sets. However, these data are in agreement with previous studies in humans suggesting that while actigraphy seems to have high accuracy for measuring sleep duration compared to EEG-based sleep recording, it may show bias when evaluating sleep latency (i.e., longer sleep latency), particularly in nights with disrupted sleep (Chinoy et al., 2021). Further studies validating the use of actigraphy in non-human primates compared to EEG-based sleep recording are warranted. It is important to note that, while actigraphy may provide an accurate measure of sleep duration, it does not allow for the investigation of sleep architecture. Studying sleep architecture allows for the identification of pharmacological treatments that either do not alter or promote physiological sleep in the absence of modifying the distribution of sleep stages across the night. This becomes particularly important when considering the present findings, in which 0.1 mg/kg methamphetamine did not alter gross sleep measures (sleep latency and duration) or actigraphy-based sleep parameters (Berro et al., 2021a), but did decrease time spent in N3 and REM sleep stages, which would still be considered sleep impairment. However, while EEG-based sleep studies provide the most direct assessment of sleep, evaluating sleep using EEG is not always practical, both in pre-clinical and in clinical studies, due to its high cost, specialized training, and time burden required to conduct and interpret these studies (Chinoy et al., 2021). Actigraphy, on the other hand, overcomes many of these barriers, and is a much more accessible model. Understanding the advantages and limitations of both models is important, and using them in a complementary manner seems to be an ideal approach to study sleep in primates.

Our findings are generally consistent with a previous study showing that oral methamphetamine administration dose-dependently disrupted sleep in recreational stimulant users (Herrmann et al., 2017). In that study, oral doses of 20 and 40 mg resulted in sleep disruption (Herrmann et al., 2017), which are doses previously shown to result in plasma levels of ∼50 to ∼100 ng/ml and to engender positive subjective effects (Kirkpatrick et al., 2012). Of note, a prior report by Banks et al. (2016) evaluated plasma methamphetamine levels after i.m. administration in rhesus monkeys and obtained an average Cmax value of 51 ng/ml at 0.32 mg/kg. Therefore, while we do not have plasma/CNS exposure levels that would allow definitive conclusions, if we assume that CNS exposure levels are approximately the same between human and rhesus monkey, then the highest dose used in the present study (0.3 mg/kg, i.m.) is consistent with those used by Herrmann et al. (2017) in recreational stimulant users. Importantly, these doses were supra-therapeutic (methamphetamine doses recommended for treatment of ADHD are 5 mg, once or twice daily), raising the possibility that at least the 0.3 mg/kg dose used in the present study reflects dose levels used by recreational methamphetamine users. At these relatively higher doses, Herrmann et al. (2017) demonstrated increased sleep latency and WASO and decreased total sleep time, reflecting diminished time spent in N2 and REM sleep stages. The main difference between our study and that of Herrmann et al. (2017) was that in the present report, methamphetamine also decreased time spent in sleep stage N3 (slow-wave sleep). While no significant changes were reported in N3 sleep duration in human methamphetamine users, a non-significant decrease in N3 sleep stage time was observed between placebo (92 ± 46 min) and the highest dose of methamphetamine (76 ± 40 min). Differences between the current study and the study by Herrmann et al. (2017) may be due to species (rhesus monkeys vs. humans), but other differences may have played a role, such as route of methamphetamine administration (oral vs. intramuscular) and stimulant use history (recreational users vs. naïve subjects).

In fact, we have previously demonstrated that a chronic history of methamphetamine intake is associated with the development of tolerance to its effects on actigraphy-based sleep in rhesus monkeys (Berro et al., 2017a). Herrmann et al. (2017) had an average of 1 week between sleep study sessions, with no stimulant use 24 h before each sleep study. Nonetheless, the relatively extensive history of stimulant use may have dampened the effects of methamphetamine on sleep (Herrmann et al., 2017). Regardless, the main effect reported in the human sleep study was a decrease in REM sleep duration by morning methamphetamine administration (Herrmann et al., 2017), an effect that was also observed in our study, even at methamphetamine doses that did not alter other sleep stages, such as N2 and N3. It is also important to note that in our previous study, interruption of drug intake by monkeys, which was designed to emulate binge patterns of human methamphetamine abuse (Ding et al., 2014), extended the deleterious effects of methamphetamine on sleep-like measures (Berro et al., 2017a). Therefore, based on the current and previous data, the pattern and frequency of typical methamphetamine use by humans, i.e., binge patterns, likely would result in persistent sleep impairments.

The most striking similarity between the present study, our previous self-administration studies (Berro et al., 2016, 2017a,b, 2021a) and the study by Herrmann et al. (2017) is that methamphetamine was administered early in the morning. While the mean elimination half-life for oral methamphetamine in humans is approximately 9 h (Shappell et al., 1996), the half-life of intramuscular methamphetamine in rhesus monkeys is approximately 3 h (Banks et al., 2016). Therefore, the fact that sleep disruption is observed 8 and 14 h after methamphetamine administration in rhesus monkeys and humans, respectively, suggests an important downstream neuroplasticity mechanism that occurs at relatively low exposure levels. Studies from our laboratory suggest that orexin-mediated mechanisms are involved in methamphetamine-induced hyperarousal and sleep disruption, and block the downstream effects of methamphetamine on actigraphy-based sleep when given as a pretreatment before morning methamphetamine administrations (Berro et al., 2021a). However, the specific mechanism by which orexin mediates these effects remains to be identified.

Previous studies have reported the effects of other stimulants, including caffeine (Authier et al., 2014; Goonawardena et al., 2018) and d-amphetamine (Authier et al., 2014), in cynomolgus monkeys using fully-implantable telemetry. Acute oral and intramuscular (i.m.) administration of caffeine immediately before “lights off” significantly decreased total sleep time by decreasing time spent in sleep stages N2 and REM (oral caffeine; Authier et al., 2014) and N3 and REM (i.m. caffeine; Goonawardena et al., 2018). Oral administration of d-amphetamine immediately before “lights off” also decreased total sleep time by decreasing N2 and REM sleep time (Authier et al., 2014). Overall, these findings are similar to our results with methamphetamine, and suggest that decreases in REM sleep duration are the most prominent and consistent effects of stimulant administration on sleep architecture, albeit *via* different mechanisms of CNS stimulant action.

Even with the marked reduction in sleep duration during the dark (inactive) phase, no sleep rebound was observed during the 24 h light and dark phases following caffeine (Goonawardena et al., 2018) or d-amphetamine (Authier et al., 2014) administration in cynomolgus monkeys. In contrast, sleep rebound effects were observed during the following day (second night) after methamphetamine administration in the present study. Impaired sleep during the night after methamphetamine administration led to an apparent compensatory increase in total sleep time reflected by a selective increase in time spent in sleep stages N3 and REM, as well as a decrease in REM sleep latency, during the following night. Importantly, those effects were only observed for the dose of methamphetamine that significantly decreased total sleep time (0.3 mg/kg), while a lower dose (0.1 mg/kg) that also induced a significant decrease in time spent in N3 and REM sleep, but not in total sleep time, did not cause significant compensatory increases in sleep duration the next day. Therefore, our findings suggest that significant sleep suppression must occur for compensatory rebound to happen, and that slow-wave (N3) and REM sleep are the first stages to be recovered following a night of sleep impairment caused by methamphetamine administration. These findings are in agreement with previous studies showing selective compensatory increases in N3 and REM sleep following sleep suppression induced by acute treatment with another drug class, the benzodiazepines (Okuma et al., 1975; Pagel and Parnes, 2001), or periods of insufficient sleep (Ferrara et al., 2000; Brillante et al., 2012; Feriante and Singh, 2021).

Some additional considerations regarding the present study are worth noting. First, our study only included male subjects, and the extent to which these findings generalize to females is unknown. We also used acute methamphetamine administrations in naïve monkeys, and as discussed above, effects of methamphetamine on sleep vary according to frequency and duration of exposure (Berro et al., 2017a). Finally, the use of non-contingent methamphetamine administration, instead of in the context of self-administration used previously (e.g., Berro et al., 2016), may limit the translational relevance of the study. However, by using experimenter-administered methamphetamine, the present findings replicate a previous study from our group showing that methamphetamine-induced disruption of actigraphy-based sleep is not dependent on the drug being available in a self-administration context (Berro et al., 2021a). Of note, the experimental design used in the present study has some unique advantages for investigating stimulant-induced sleep impairment. For example, by administering methamphetamine non-contingently, we were able to standardize the methamphetamine dosing, which is a challenge in self-administration studies, as the animals control drug intake with contingent drug delivery. The present study also replicates our previous finding showing that methamphetamine disrupts sleep, even in the absence of drug-associated cues, which also contribute to methamphetamine-induced sleep impairment in the context of drug self-administration (Berro et al., 2016).

In summary, our findings show that morning methamphetamine administration produces significant and dose-dependent disruptions in sleep duration and architecture in rhesus monkeys, even when administered 8 h before the inactive (dark) phase. These findings are in agreement with actigraphy-based sleep studies in monkeys (Berro et al., 2016, 2017a,b) and a polysomnography study in humans (Herrmann et al., 2017). Therefore, individuals who use methamphetamine are at risk for the development of sleep impairment and are also likely to experience negative health outcomes associated with sleep deprivation (for review, see Li et al., 2021). Similar effects have been reported in macaques with other stimulants (Authier et al., 2014; Goonawardena et al., 2018), with REM sleep suppression being the most marked and consistent effect of stimulant administration. Thus, sleep evaluations are critical in individuals who use and misuse stimulants, including prescription stimulants. Importantly, individuals with a history of sleep problems are at higher risk for the development of substance use disorders and are more likely to relapse following treatment (Brower and Perron, 2010; Wong et al., 2015; Roehrs et al., 2021), further emphasizing the importance of sleep management before stimulant prescription, as well as in the treatment of stimulant use disorders.

## Data Availability Statement

The raw data supporting the conclusions of this article will be made available by the authors, without undue reservation.

## Ethics Statement

The animal study was reviewed and approved by Institutional Animal Care and Use Committee of the University of Mississippi Medical Center.

## Author Contributions

LB and JR were responsible for the study concept and design. LB contributed to the acquisition of data and drafted the manuscript. All authors assisted with data analysis and interpretation of findings and provided critical revision of the manuscript for important intellectual content and approved the final version for publication.

## Conflict of Interest

The authors declare that the research was conducted in the absence of any commercial or financial relationships that could be construed as a potential conflict of interest.

## Publisher’s Note

All claims expressed in this article are solely those of the authors and do not necessarily represent those of their affiliated organizations, or those of the publisher, the editors and the reviewers. Any product that may be evaluated in this article, or claim that may be made by its manufacturer, is not guaranteed or endorsed by the publisher.

## References

[B1] Authier S. Bassett L. Pouliot M. Rachalski A. Troncy E. Paquette D. (2014). Effects of amphetamine, diazepam and caffeine on polysomnography (EEG, EMG, EOG)-derived variables measured using telemetry in Cynomolgus monkeys. *J. Pharmacol. Toxicol. Methods* 70 86–93. 10.1016/j.vascn.2014.05.003 24878255

[B2] Banks M. L. Smith D. A. Kisor D. F. Poklis J. L. (2016). Relationship between discriminative stimulus effects and plasma methamphetamine and amphetamine levels of intramuscular methamphetamine in male rhesus monkeys. *Pharmacol. Biochem. Behav.* 141 58–65. 10.1016/j.pbb.2015.12.001 26656213 PMC4724286

[B3] Bassetti C. L. A. Adamantidis A. Burdakov D. Han F. Gay S. Kallweit U. (2019). Narcolepsy - clinical spectrum, aetiopathophysiology, diagnosis and treatment. *Nat. Rev. Neurol.* 15 519–539. 10.1038/s41582-019-0226-9 31324898

[B4] Berro L. F. Andersen M. L. Howell L. L. (2017a). Assessment of tolerance to the effects of methamphetamine on daytime and nighttime activity evaluated with actigraphy in rhesus monkeys. *Psychopharmacology* 234 2277–2287. 10.1007/s00213-017-4654-1 28589263 PMC5522354

[B5] Berro L. F. Andersen M. L. Tufik S. Howell L. L. (2017b). GABA(A) receptor positive allosteric modulators modify the abuse-related behavioral and neurochemical effects of methamphetamine in rhesus monkeys. *Neuropharmacology* 123 299–309. 10.1016/j.neuropharm.2017.05.010 28495376 PMC5513762

[B6] Berro L. F. Andersen M. L. Tufik S. Howell L. L. (2016). Actigraphy-based sleep parameters during the reinstatement of methamphetamine self-administration in rhesus monkeys. *Exp. Clin. Psychopharmacol.* 24 142–146. 10.1037/pha0000064 26882419 PMC4795967

[B7] Berro L. F. Moreira-Junior E. D. C. Rowlett J. K. (2021a). The dual orexin receptor antagonist almorexant blocks the sleep-disrupting and daytime stimulant effects of methamphetamine in rhesus monkeys. *Drug Alcohol Depend* 227:108930. 10.1016/j.drugalcdep.2021.108930 34358767 PMC8464508

[B8] Berro L. F. Overton J. S. Reeves-Darby J. A. Rowlett J. K. (2021b). Alprazolam-induced EEG spectral power changes in rhesus monkeys: a translational model for the evaluation of the behavioral effects of benzodiazepines. *Psychopharmacology* 238 1373–1386. 10.1007/s00213-021-05793-z 33594504 PMC8177744

[B9] Berro L. F. Pareek T. Reeves-Darby J. A. Andersen M. L. Howell L. L. Platt D. M. (2022). Influence of Pair-housing on Sleep Parameters Evaluated with Actigraphy in Female Rhesus Monkeys. *J. Am. Assoc. Lab Anim. Sci.* 10.30802/AALAS-JAALAS-21-000027 [Epub online ahead of print]. 35012705 PMC8956211

[B10] Berry R. B. Albertario C. L. Harding S. M. Lloyd R. M. Plante D. T. Quan S. F. (2018). *The AASM Manual for the Scoring of Sleep and Associated Events: Rules, Terminology and Technical Specifications. Version 2.5.* Darien, IL: American Academy of Sleep Medicine.

[B11] Boulos M. I. Jairam T. Kendzerska T. Im J. Mekhael A. Murray B. J. (2019). Normal polysomnography parameters in healthy adults: a systematic review and meta-analysis. *Lancet Respir. Med.* 7 533–543. 10.1016/S2213-2600(19)30057-8 31006560

[B12] Brillante R. Cossa G. Liu P. Y. Laks L. (2012). Rapid eye movement and slow-wave sleep rebound after one night of continuous positive airway pressure for obstructive sleep apnoea. *Respirology* 17 547–553. 10.1111/j.1440-1843.2012.02147.x 22309157

[B13] Brower K. J. Perron B. E. (2010). Sleep disturbance as a universal risk factor for relapse in addictions to psychoactive substances. *Med. Hypotheses.* 74 928–933. 10.1016/j.mehy.2009.10.020 19910125 PMC2850945

[B14] Chinoy E. D. Cuellar J. A. Huwa K. E. Jameson J. T. Watson C. H. Bessman S. C. (2021). Performance of seven consumer sleep-tracking devices compared with polysomnography. *Sleep* 44 zsaa291. 10.1093/sleep/zsaa291 33378539 PMC8120339

[B15] Compton W. M. Han B. Blanco C. Johnson K. Jones C. M. (2018). Prevalence and correlates of prescription stimulant use, misuse, use disorders, and motivations for misuse among adults in the United States. *Am. J. Psychiatr.* 175 741–755. 10.1176/appi.ajp.2018.17091048 29656665 PMC6070393

[B16] Cruickshank C. C. Dyer K. R. (2009). A review of the clinical pharmacology of methamphetamine. *Addiction* 104 1085–1099. 10.1111/j.1360-0443.2009.02564.x 19426289

[B17] Daley J. T. Turner R. S. Freeman A. Bliwise D. L. Rye D. B. (2006). Prolonged assessment of sleep and daytime sleepiness in unrestrained *Macaca mulatta*. *Sleep* 29 221–231. 16494090

[B18] Ding Y. Lin H. Zhou L. Yan H. He N. (2014). Adverse childhood experiences and interaction with methamphetamine use frequency in the risk of methamphetamine-associated psychosis. *Drug Alcohol Depend.* 142 295–300. 10.1016/j.drugalcdep.2014.06.042 25064022

[B19] Feriante J. Singh S. (2021). *REM Rebound Effect. In: StatPearls [Internet.* Treasure Island (FL): StatPearls Publishing. 32809548

[B20] Ferrara M. De Gennaro L. Casagrande M. Bertini M. (2000). Selective slow-wave sleep deprivation and time-of-night effects on cognitive performance upon awakening. *Psychophysiology* 37 440–446. 10934902

[B21] Goonawardena A. V. Morairty S. R. Orellana G. A. Willoughby A. R. Wallace T. L. Kilduff T. S. (2018). Electrophysiological characterization of sleep/wake, activity and the response to caffeine in adult cynomolgus macaques. *Neurobiol. Sleep Circad. Rhythms* 6 9–23. 10.1016/j.nbscr.2018.08.001 31236518 PMC6586594

[B22] Herbeck D. M. Brecht M. L. Lovinger K. (2015). Mortality, causes of death, and health status among methamphetamine users. *J. Addict. Dis.* 34 88–100. 10.1080/10550887.2014.975610 25415384 PMC4684255

[B23] Herrmann E. S. Johnson P. S. Bruner N. R. Vandrey R. Johnson M. W. (2017). Morning administration of oral methamphetamine dose-dependently disrupts nighttime sleep in recreational stimulant users. *Drug Alcohol Depend* 178 291–295. 10.1016/j.drugalcdep.2017.05.013 28686987

[B24] Hsieh K. C. Robinson E. L. Fuller C. A. (2008). Sleep architecture in unrestrained rhesus monkeys (*Macaca mulatta*) synchronized to 24-hour light-dark cycles. *Sleep* 31 1239–1250. 18788649 PMC2542979

[B25] Kirkpatrick M. G. Gunderson E. W. Perez A. Y. Haney M. Foltin R. W. Hart C. L. (2012). A direct comparison of the behavioral and physiological effects of methamphetamine and 3,4-methylenedioxymethamphetamine (MDMA) in humans. *Psychopharmacology* 219 109–122. 10.1007/s00213-011-2383-4 21713605 PMC4430833

[B26] Li J. Cao D. Huang Y. Chen Z. Wang R. Dong Q. (2021). Sleep duration and health outcomes: an umbrella review. *Sleep Breath* 10.1007/s11325-021-02458-1 Epub online ahead of print]. 34435311

[B27] McElroy S. L. (2017). Pharmacologic Treatments for Binge-Eating Disorder. *J. Clin. Psychiatr.* 78 14–19. 10.4088/JCP.sh16003su1c.03 28125174

[B42] National Research Council (2011). *Guide for the Care and Use of Laboratory Animals: Eighth Edition*. Washington, DC: The National Academies Press. 10.17226/12910

[B28] Okuma T. Hata N. Fujii S. (1975). Differential effects of chlorpromazine, imipramine, nitrazepam and amobarbital on REM sleep and REM density in man. *Folia. Psychiatr. Neurol. Jpn.* 29 25–37. 10.1111/j.1440-1819.1975.tb02320.x 169190

[B29] Pagel J. F. Parnes B. L. (2001). Medications for the Treatment of Sleep Disorders: An Overview. *Prim. Care Companion J. Clin. Psychiatr.* 3 118–125. 10.4088/pcc.v03n0303 15014609 PMC181172

[B30] Posner J. Polanczyk G. V. Sonuga-Barke E. (2020). Attention-deficit hyperactivity disorder. *Lancet* 395 450–462. 10.1016/S0140-6736(19)33004-1 31982036 PMC7880081

[B31] Rachalski A. Authier S. Bassett L. Pouliot M. Tremblay G. Mongrain V. (2014). Sleep electroencephalographic characteristics of the Cynomolgus monkey measured by telemetry. *J. Sleep Res.* 23 619–627. 10.1111/jsr.12189 25109588

[B32] Roehrs T. Sibai M. Roth T. (2021). Sleep and alertness disturbance and substance use disorders: A bi-directional relation. *Pharmacol. Biochem. Behav.* 203:173153. 10.1016/j.pbb.2021.173153 33582097 PMC7996967

[B33] Roffwarg H. P. Muzio J. N. Dement W. C. (1966). Ontogenetic development of the human sleep-dream cycle. *Science* 152 604–619. 10.1126/science.152.3722.604 17779492

[B34] Rommel N. Rohleder N. H. Wagenpfeil S. Haertel-Petri R. Kesting M. R. (2015). Evaluation of methamphetamine-associated socioeconomic status and addictive behaviors, and their impact on oral health. *Addict. Behav.* 50 182–187. 10.1016/j.addbeh.2015.06.040 26151583

[B35] Ruehland W. R. O’Donoghue F. J. Pierce R. J. Thornton A. T. Singh P. Copland J. M. (2011). The 2007 AASM recommendations for EEG electrode placement in polysomnography: impact on sleep and cortical arousal scoring. *Sleep* 34, 73–81. 10.1093/sleep/34.1.73 21203376 PMC3001799

[B36] Shappell S. A. Kearns G. L. Valentine J. L. Neri D. F. DeJohn C. A. (1996). Chronopharmacokinetics and chronopharmacodynamics of dextromethamphetamine in man. *J. Clin. Pharmacol.* 36 1051–1063. 10.1177/009127009603601109 8973994

[B37] Substance Abuse and Mental Health Services Administration [SAMSHA]. (2019). *Key substance use and mental health indicators in the United States: Results from the 2018 National Survey on Drug Use and Health (HHS Publication No. PEP19–5068, NSDUH Series H-54).* Rockville, MD: Center for Behavioral Health Statistics and Quality, Substance Abuse and Mental Health Services Administration.

[B38] Trotti L. M. Arnulf I. (2021). Idiopathic Hypersomnia and Other Hypersomnia Syndromes. *Neurotherapeutics* 18 20–31. 10.1007/s13311-020-00919-1 32901432 PMC8116415

[B39] Vivolo-Kantor A. M. Hoots B. E. Seth P. Jones C. M. (2020). Recent trends and associated factors of amphetamine-type stimulant overdoses in emergency departments. *Drug Alcohol Depend.* 216:108323. 10.1016/j.drugalcdep.2020.108323 33032064 PMC7606828

[B40] Vrajová M. Šlamberová R. Hoschl C. Ovsepian S. V. (2021). Methamphetamine and sleep impairments: neurobehavioral correlates and molecular mechanisms. *Sleep.* 44:zsab001. 10.1093/sleep/zsab001 33406259

[B41] Wong M. M. Robertson G. C. Dyson R. B. (2015). Prospective relationship between poor sleep and substance-related problems in a national sample of adolescents. *Alcohol. Clin. Exp. Res.* 39 355–362. 10.1111/acer.12618 25598438 PMC4331208

